# Radiotherapy after mastectomy has significant survival benefits for inflammatory breast cancer: a SEER population-based retrospective study

**DOI:** 10.7717/peerj.8512

**Published:** 2020-02-03

**Authors:** Zhi-wen Li, Miao Zhang, Yong-jing Yang, Zi-jun Zhou, Yan-ling Liu, Hang Li, Bo Bao, Jian-dong Diao, Dun-wei Wang

**Affiliations:** 1 Department of Anesthesiology, First Hospital of Jilin University, Changchun, Jilin, China; 2 Departments of Radiation Oncology, Jilin Cancer Hospital, Changchun, Jilin, China; 3 Department of Breast Surgery, Jilin Cancer Hospital, Changchun, Jilin, China; 4 Departments of Oncology, Jilin Cancer Hospital, Changchun, Jilin, China; 5 Department of Thoracic Surgery, Jilin Cancer Hospital, Changchun, Jilin, China; 6 Department of Oncology and Hematology, China-Japan Union Hospital of Jilin University, Changchun, Jilin, China

**Keywords:** Cancer-specific survival, Inflammatory breast cancer, Radiotherapy, Overall survival, SEER database

## Abstract

**Objectives:**

The survival benefit of postmastectomy radiotherapy (PMRT) has not been fully proven in inflammatory breast cancer (IBC). Thus, in the present research, we aimed at elucidating the effects of PMRT on the survival of IBC patients.

**Methods:**

Eligible patients were collected from the Surveillance, Epidemiology, and End Results (SEER) dataset between 2010 and 2013. The Kaplan-Meier method along with the log-rank test was utilized for the comparison of both the overall survival (OS) andthe cancer-specific survival (CSS) in patients undergoing PMRT or not. Additionally, multivariate survival analysis of CSS and OS were performed using the Cox proportional hazard model.

**Results:**

In total, 293 eligible cases were identified, with the median follow-up time of 27 months (range: 5–59 months). After propensity score matching (PSM), 188 patients (94 for each) were classified intothe No-PMRT and the PMRT group. Consequently, significantly higher OS rates were detected in the PMRT group compared with the No-PMRT group prior to PSM (*P* = 0.034), and significantly higher CSS (*P* = 0.013) and OS (*P* = 0.0063) rates were observed following PSM. Furthermore, multivariate analysis revealed thatPMRT [CSS (HR: 0.519, 95% CI [0.287–0.939], *P* = 0.030); OS (HR: 0.480, 95% CI [0.269–0.859], *P* = 0.013)], as well as Her2+/HR+ subtype, was independent favorable prognostic factors.Besides, black ethnicity, AJCC stage IV and triple-negative subtype were independent unfavorable prognostic factors. Further subgroup analysis revealed that most of the study population could benefit from PMRT, no matter OS or CSS.

**Conclusions:**

Our findings support that PMRT could improve the survival of IBC patients.

## Introduction

Inflammatory breast cancer (IBC) is a rare and aggressive clinicopathological entity of breast cancer, representing 1% to 6% of all types of breast cancer (Hance et al., 2005). In the tumor-node-metastasis (TNM) stage classification of breast cancer, IBC is categorized as T4d and clinically featured by the diffused duration of the skin with an erysipeloid edge, generally without underlying mass (Kleer, Van Golen & Merajver, 2000). Due to its rare incidence, IBC data is mainly acquired from small, single-centered, retrospective research, or extrapolated from randomized prospective studies or clinical experience of non-IBC patients (Van Uden et al., 2015).

Historically, IBC was treated by surgery and/or radiotherapy. The combination of surgery and radiotherapy leads to the improvement of overall survival (Chang, Buzdar & Hursting, 1998). Radiotherapy is considered as the most effective local therapeutic modality only after surgery. However, the application of radiotherapy in IBC, optionally along with concurrent chemotherapy, is generally recommended only if radical resection (R0) is not achievable following primary systemic therapy (Bartelink et al., 1997; Bourgier et al., 2012; Van Uden et al., 2017). Notably, the great advance in modern and more effective neoadjuvant therapy has enhanced the partial or complete clinical response rate, and additionally, a considerable percentage of the previously unresectable subjects are proper for mastectomy (Baldini et al., 2004; Gianni et al., 2010). However, the survival benefit for postmastectomy radiotherapy (PMRT) has not been fully validated up to now (Orecchia, 2018). Current guidelines and recommendations for treatment of IBC stated that aggressive trimodality treatment, including PMRT, can be recommended, regardless of the response to systemic treatment (Budach et al., 2015; Dawood et al., 2011). In addition, PMRT is under used in clinical practice (Loveland-Jones et al., 2016). Therefore, it is urgent to precisely assess the prognostic role of PMRT in IBC.

In this research, a large cohort of IBC cases was selected from the Surveillance, Epidemiology, and End Results (SEER) dataset, followed by an investigation of the survival differences between PMRT and No-PMRT groups. We aimed at assessing factors correlated with the prognosis of IBC patients, highlighting the effects of PMRT on the outcomes of IBC patients.

## Material and Methods

### Ethics statement

The National Cancer Institute’s SEER program, initiated from 1973 and annually updated, uses population-based data to develop comprehensive sources (Duggan et al., 2016). It covers approximately 30% of the US population throughout several geographic regions (Cronin, Ries & Edwards, 2014). The SEER Research Data Agreement was signed for accessing SEER information with the use of reference number 16462-Nov2016. Additionally, the research methods were performed to obtain data following approved guidelines. The data analysis was considered by the Office for Human Research Protection to be non-human subjects who were researched by the United States Department of Health and Human Services, as they were publicly available and de-identified. Thus, no approval was required by the institutional review board.

### Study population

Patient data were acquired using the SEER database (Submission, November 2016). The SEER*State v8.3.5 tool, released on March 6, 2018, was used to determine and select eligible patients. Additionally, the study duration ranged from 2010 to December 2013. The inclusion criteria were as follows: (1) only primary female IBC patients undergoing mastectomy were selected; (2) IBC was diagnosed in line with the International Classification of Disease for Oncology, Third Edition (ICD-O-3; coded as 8530/3). The exclusion criteria were listed in the following: (1) patients were burdened with multiple primary tumors; (2) patients were only clinically diagnosed; (3) patients without mastectomy; (4) patients with preoperative radiotherapy or unknown sequence of radiotherapy; (5) patients with incomplete important clinicopathological information, including age at diagnosis, race, 7th AJCC tumor stage, and surgical style; (6) patients died within 3 months after surgery; (7) patients withoutprognostic information. The remaining patients were enrolled as SEER primary cohort.

### Covariates and endpoints

Patients’ characteristics were analyzed according to the following 9 factors: age (<40, 40–60, or >60 years), race (white, black, or other), marital status (married, unmarried, unknown), tumor grade (Grade I/II, Grade III/IV, unknown), AJCC stage (IIIA, IIIB, IV), breast Subtype (Her2-/hormone receptor (HoR)+, Her2+/HoR-, Her2+/HoR+, Triple Negative, unknown); surgery (simple mastectomy, radical mastectomy), chemotherapy (no/unknown, yes), PMRT (no/unknown, yes). The widowed or single (having a domestic partner or never married) or separated or divorced were classified as unmarried. Age was further transformed into a categorical variable according to recognized cut-off values. Race was divided into white, black and other (including Asian/Pacific Islander, American Indian/Alaska native, and unknown). A simple mastectomy removes all breast tissue, the nipple, and areolar complex. An axillary dissection is not done. A radical mastectomy removes all breast tissue, the nipple, the areolar complex, and variable amounts of breast skin, which includes axillary lymph node dissection. In addition, all eligible subjects were restaged in accordance with the 8th AJCC TNM staging system. The HoR status of tumor was stratified into HoR-positive [ER+/PR+, ER-/PR+, and ER+/PR-] and HoR-negative (ER-/PR-). The definition of ER/PR+ disease was ofor over 1% cells stained positive (Howlader et al., 2014). Our research was limited to between 2010 and2013 because breast subtype was published from 2010 in the SEER database.

The primary endpoints in this research were overall survival (OS) as well as cancer-specific survival (CSS). CSS was defined as the interval from diagnosis to the last follow-up or death caused by IBC.OS was defined as the duration from diagnosis to the last follow-up or death. There was a predetermined cut-off date based on the SEER 2016 submission database, containing death information until 2014. Therefore, a cut-off date of December 31, 2014 was utilized in our study.

### Propensity score matching (PSM)

In observational research, selection bias is unavoidable, leading to unevenly distributed confounding factors between two groups. A PSM is defined as the conditional possibility of assignment to a certain treatment given a vector of observed covariates (Little & Rubin, 2000). Thus, in order to decrease selection bias as well as unevenly distributed confounding factors, the PSM method was employed in this research (Pattanayak, Rubin & Zell, 2011). The logistic regression model was used to estimate propensity scores of all patients by enrolling all covariates potentially affecting patient survival, followed by the PSM plug-in of the SPSS software to estimate the propensity score of every case. PSM was performed afterward using 1:1 nearest-neighbor matching with a caliper of0.05 to obtain a matched pair. PSM was conducted between the PMRT group and the No-PMRT group of each subgroup.

### Statistical analyses

Pearson’s *χ*2 or Fisher’s exact tests were utilized for comparison of clinicopathologic characteristics before and after PSM. Kaplan–Meier (K-M) method was utilized for estimation of the univariate analysis, along with the log-rank test to assess the differences of CSS and OS. Moreover, the risk of mortality was assessed by multivariate Cox proportional hazards regression models, which were also used for subgroup analyses. Variables with potential significance in the univariate analysis (*P* < 0.05) or clinically regarded as prognostic factors were further enrolled in multivariate analysis. All statistical analyses were conducted using SPSS software (SPSS Inc., Chicago, USA, version 19.0). Graph Pad Prism 5 was employed to generate the survival curve and forest plots. A two-sided *P* < 0.05 was considered as statistically significant.

## Results

### Patient characteristics before and after PSM

**Figure 1:**
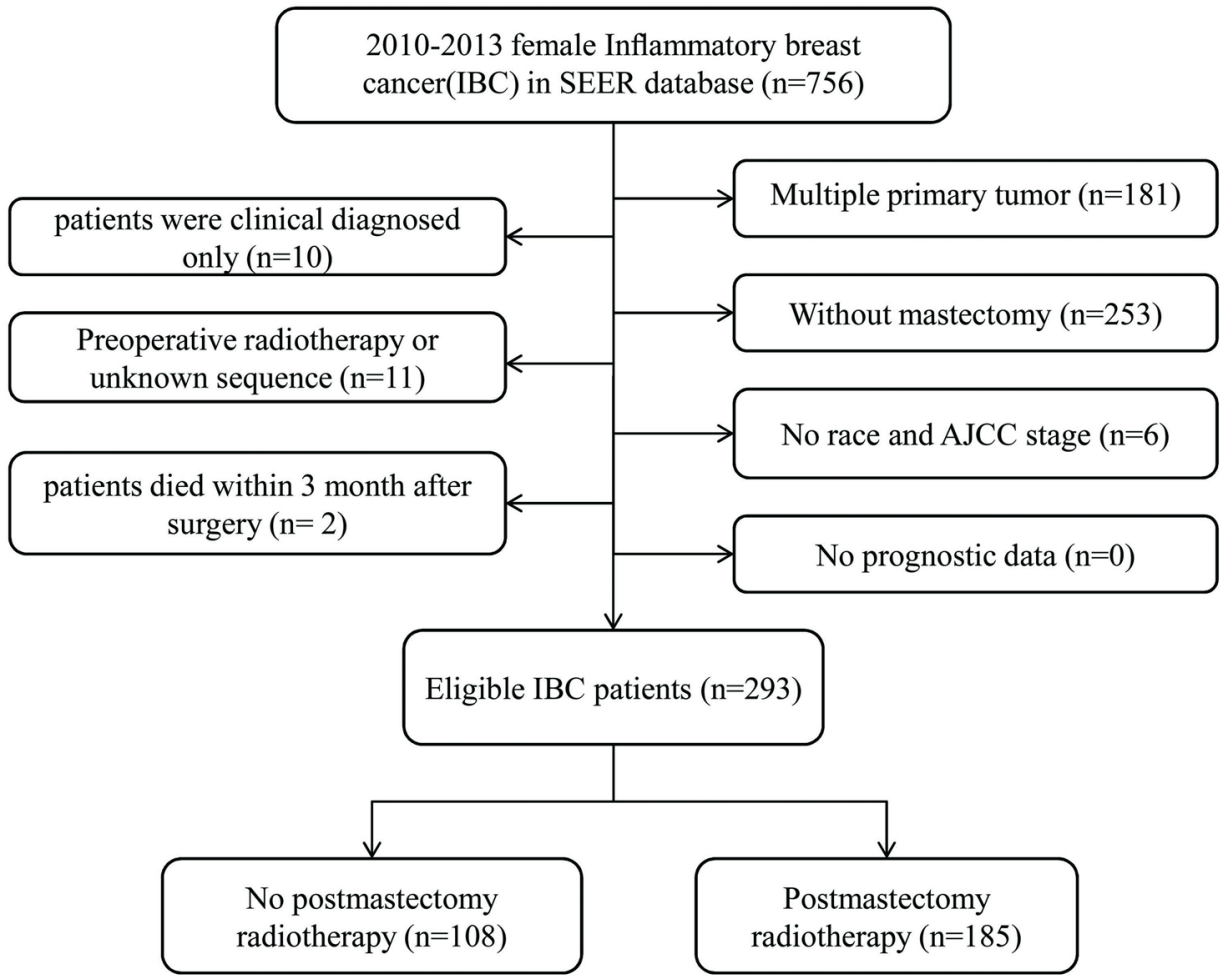
Flow chart for screening eligible patients.

In total, 293 cases were enrolled in this research. According to whether the patients received PMRT, two groups were defined: No-PMRT group (*n* = 108) and PMRT group (*n* = 185).The specific screening process was shown in Fig. 1. Patient characteristics in both cohorts were presented in Table 1. Only two patients had bilateral breast cancer. The median follow-up duration of all subjects was 27 months (5–59 months).The following variables, age, tumor grade, race, marital status, AJCC stage, breast subtype; surgery style and chemotherapy were included in PSM. The plot of the propensity distribution score was shown in File S1. Specifically, the PMRT group had a higher proportion of married and received chemotherapy before PSM. After PSM at a 1:1 ratio, there were 94 patients in each group, and all the baseline characteristics of the two groups were balanced.

**Table 1:** Baseline characteristics before and after propensity score matching (PSM).

Variables	Before PSM			After PSM		
	No-PMRT (*n* = 108)	PMRT (*n* = 185)	*P*	No-PMRT (*n* = 94)	PMRT (*n* = 94)	*P*
Age			0.921			0.483
<40	14 (12.96%)	27 (14.59%)		11 (11.70%)	11 (11.70%)	
40–60	59 (54.63%)	98 (52.97%)		56 (59.57%)	63 (67.02%)	
>60	35 (32.41%)	60 (32.43%)		27 (28.72%)	20 (21.28%)	
Race			0.507			0.270
White	82 (75.93%)	151 (81.62%)		69 (73.40%)	78 (82.98%)	
Black	16 (14.81%)	21 (11.35%)		16 (17.02%)	11 (11.70%)	
Other	10 (9.26%)	13 (7.03%)		9 (9.57%)	5 (5.32%)	
Marital status			0.007			0.127
Married	44 (40.74%)	109 (58.92%)		40 (42.55%)	53 (56.38%)	
Unmarried	56 (51.85%)	70 (37.84%)		46 (48.94%)	37 (39.36%)	
Unknown	8 (7.41%)	6 (3.24%)		8 (8.51%)	4 (4.26%)	
Grade			0.081			0.079
Grade I/II	24 (22.22%)	51 (27.57%)		19 (20.21%)	30 (31.91%)	
Grade III/IV	69 (63.89%)	94 (50.81%)		60 (63.83%)	45 (47.87%)	
Unknown	15 (13.89%)	40 (21.62%)		15 (15.96%)	19 (20.21%)	
AJCC stage			0.088			0.082
IIIA	62 (57.41%)	124 (67.03%)		52 (55.32%)	61 (64.89%)	
IIIB	17 (15.74%)	31 (16.76%)		13 (13.83%)	17 (18.09%)	
IV	29 (26.85%)	30 (16.22%)		29 (30.85%)	16 (17.02%)	
Breast Subtype			0.976			0.805
Her2-/HR+	37 (34.26%)	65 (35.14%)		30 (31.91%)	31 (32.98%)	
Her2+/HR-	17 (15.74%)	32 (17.30%)		15 (15.96%)	11 (11.70%)	
Her2+/HR+	22 (20.37%)	38 (20.54%)		20 (21.28%)	26 (27.66%)	
Triple Negative	25 (23.15%)	41 (22.16%)		23 (24.47%)	20 (21.28%)	
Unknown	7 (6.48%)	9 (4.86%)		6 (6.38%)	6 (6.38%)	
Surgery			0.069			0.154
Simple Mastectomy	33 (30.56%)	39 (21.08%)		24 (25.53%)	16 (17.02%)	
Radical mastectomy	75 (69.44%)	146 (78.92%)		70 (74.47%)	78 (82.98%)	
Chemotherapy			<0.001			1.000
No/unknown	16 (14.81%)	3 (1.62%)		3 (3.19%)	3 (3.19%)	
Yes	92 (85.19%)	182 (98.38%)		91 (96.81%)	91 (96.81%)	

**Notes.**

Abbreviations HoR Hormone receptor PSM propensity score matching PMRT postmastectomy radiotherapy

### CSS and OS of patients before and after PSM

**Figure 2:**
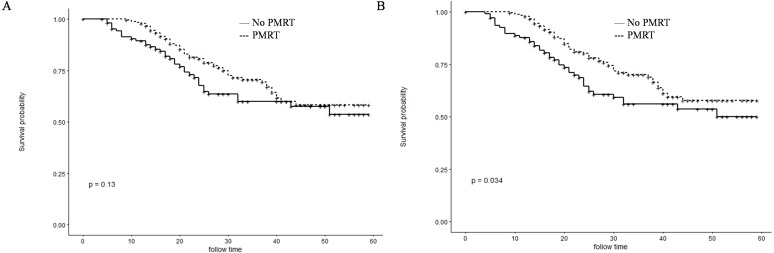
Kaplan–Meier curves for CSS and OS before PSM. Cancer-specific survival (A) and overall survival (B) differences between postmastectomy radiotherapy (PMRT) and No-PMRT groups, before 1:1 Propensity Score Matching analysis. *P* = 0.13 for PMRT group compared to No-PMRT group for CSS; *P = 0.034 for PMRT group compared to No-PMRT group for OS.

**Figure 3:**
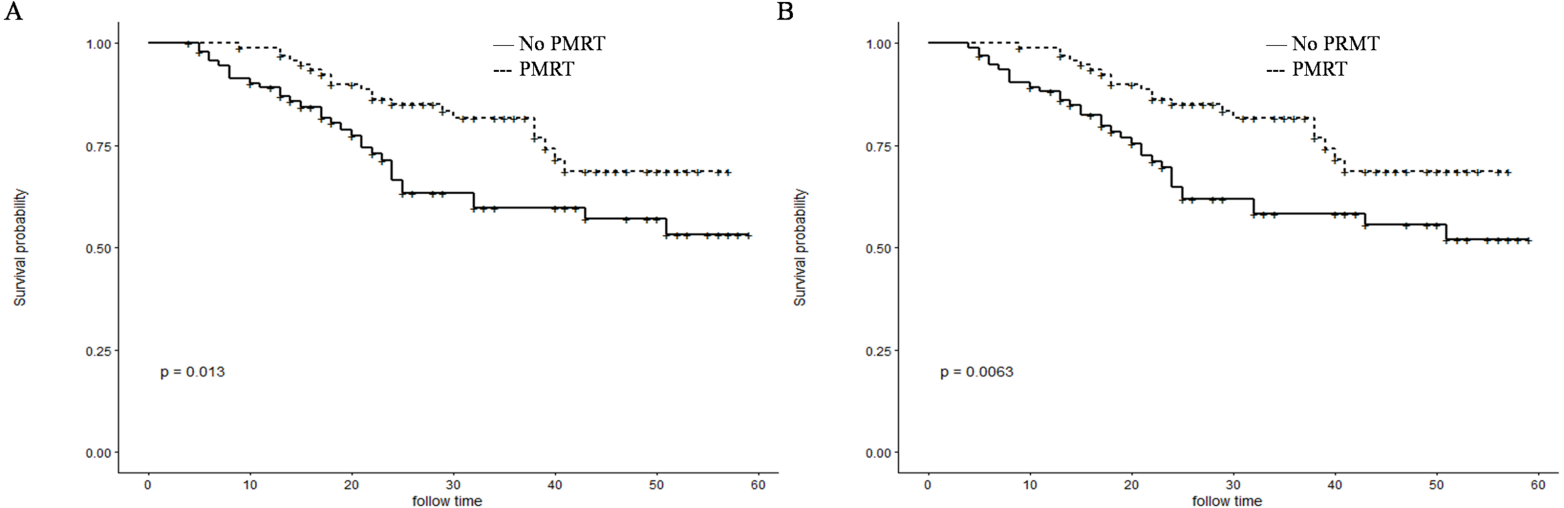
Kaplan–Meier curves for CSS and OS after PSM. Cancer-specific survival (A) and overall survival (B) differences between postmastectomy radiotherapy (PMRT) and No-PMRT groups, after 1:1 Propensity Score Matching analysis. *P = 0.013 for PMRT group compared to No-PMRT group for CSS; **P = 0.0063 for PMRT group compared to No-PMRT postmastectomy RT (PMRT) group for OS.

Before PSM, patients in the PMRT group showed significantly higher 1- and 3-year OS rates than those in the No-PMRT group (97.8%, 70.2%, respectively vs. 87.8% and 56.0%, respectively; *P* = 0.034) (Fig. 2B). However, there was no difference of1-and 3-year CSS between the two groups (97.8%, 70.1%, respectively vs. 89.5%, 60.1%, respectively; *P* = 0.13) (Fig. 2A). After PSM, patients in the PMRT group harbored significantly higher 1- and 3-year CSS and OS rates [CSS (99.0%, 81.8% vs. 89.1% and 59.7%, respectively, *P* = 0.013); OS (99.0%, 81.8% vs. 88.2% and 58.2%, respectively, *P* = 0.0063)] (Fig. 3).

### Prognostic factors for survival after PSM

Multivariate analysis of CSS and OS revealed that black ethnicity[CSS (HR:2.251, 95% CI [1.186–4.271], *P* = 0.029); OS (HR:2.076, 95% CI [1.102–3.912], *P* = 0.024)], AJCC stage IV [CSS (HR:4.837, 95% CI [2.548–9.180], *P* < 0.001); OS (HR:4.475, 95% CI [2.387–8.389])] and triple negative subtype [CSS (HR:3.387, 95% CI [1.697–6.762], *P* = 0.001); OS (HR:3.188, 95% CI [1.630–6.235], *P* = 0.001)] were independent unfavorable prognostic factors, and Her2+/HR+ subtype [CSS (HR:0.244, 95% CI [0.070–0.857], *P* = 0.028); OS (HR:0.225, 95% CI [0.065–0.785], *P* = 0.019)] and PMRT [CSS (HR:0.519, 95% CI [0.287–0.939], *P* = 0.030); OS (HR:0.480, 95% CI [0.269–0.859], *P* = 0.013)] were independent favorable prognostic factors (Table 2).

### Subgroup analysis for OS and CSS after PSM

**Figure 4:**
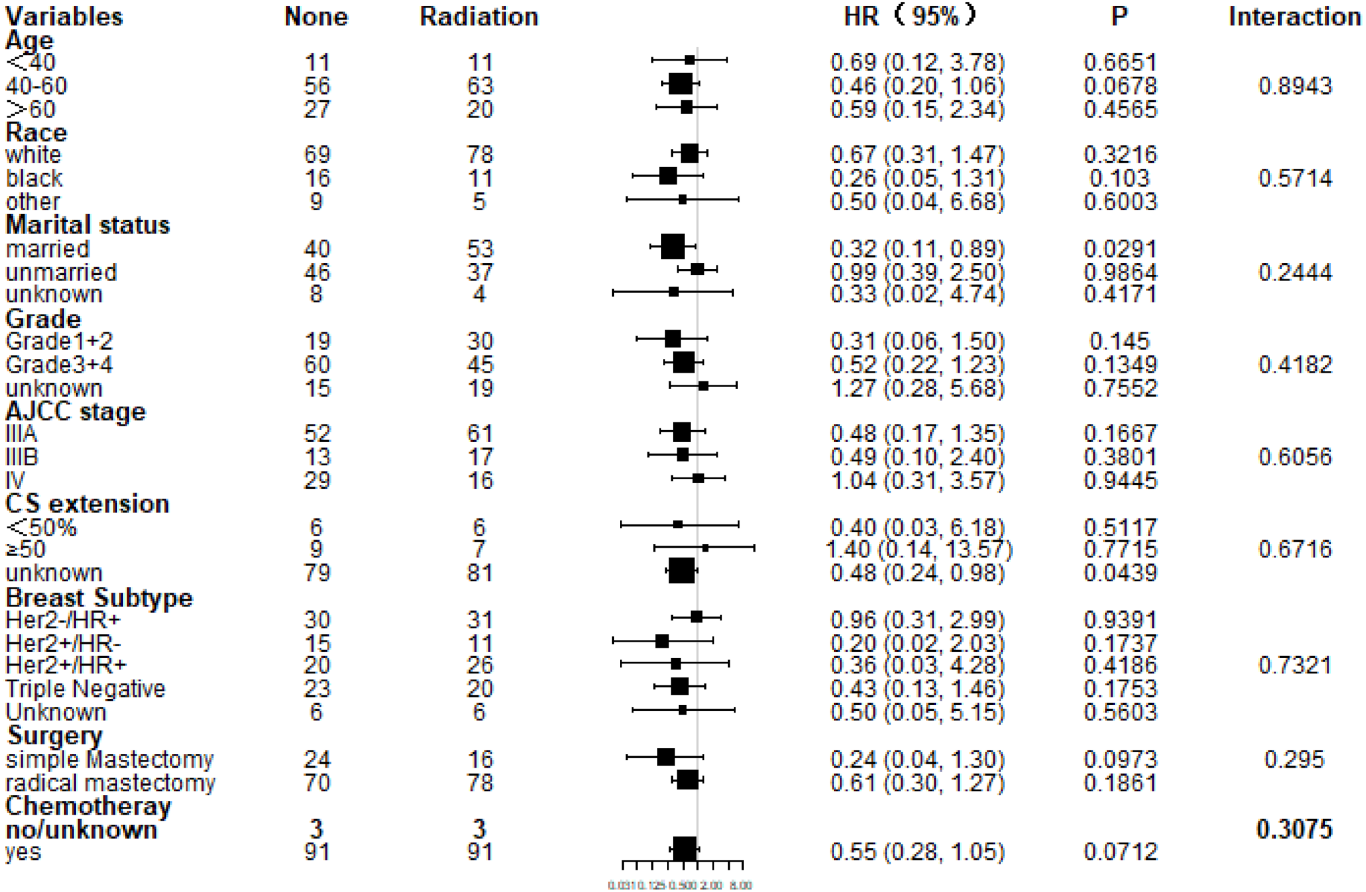
Forest plot of subgroup analysis for CSS after PSM. Subgroup analysis of cancer-specific survival between postmastectomy radiotherapy (PMRT) and No-PMRT groups, after 1:1 Propensity Score Matching analysis. Nearly all subgroups derived CSS benefit in favor of PMRT, except AJCC stage IV and CS extension ≥50%.

**Figure 5:**
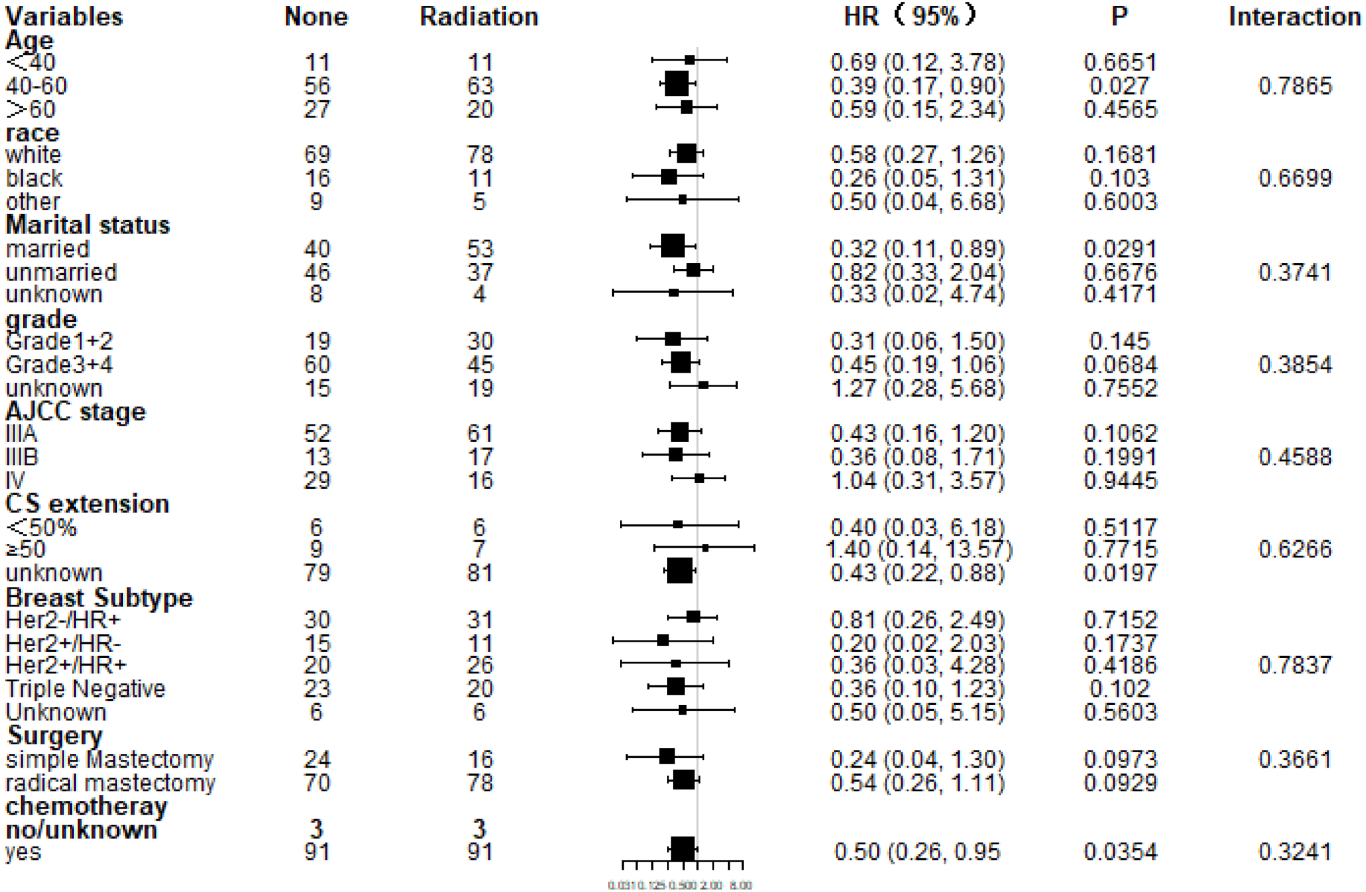
Forest plot of subgroup analysis for OS after PSM. Subgroup analysis of OS between postmastectomy radiotherapy (PMRT) and No-PMRT groups, after 1:1 Propensity Score Matching analysis. Nearly all subgroups derived OS benefit in favor of PMRT group, except AJCC stage IV and CS extension ≥50%.

Subgroup analysis of CSS and OS demonstrated that nearly all study population could benefit survival from PMRT except those with stage IV (Figs. 4 and 5). Married patients could obtain significant benefit from PMRT in terms of CSS (HR: 0.32, 95% CI [0.11–0.89], *P* = 0.029). In addition, patients who aged 40–60 (HR: 0.39, 95% CI [0.17–0.90], *P* = 0.027), married (HR: 0.32, 95% CI [0.11–0.89], *P* = 0.029), and chemotherapy (HR: 0.50, 95% CI [0.26–0.95], *P* = 0.035) also acquired significant benefit from PMRT, in terms of OS.

**Table 2:** Univariate and multivariate analyses of cancer special survival (CSS) and overall survival (OS) after propensity score matching (*N* = 188).

Variables	CSS			OS		
	Univariate analysis	Multivariate analysis		Univariate analysis	Multivariate analysis	
	*P*-value	HR (95% CI)	*P*-value	*P*-value	HR (95% CI)	*P*-value
Age	0.407		NI	0.491		NI
<40						
40–60						
>60						
Race	0.002		0.029	0.003		0.046
White		Reference			Reference	
Black		2.251 (1.186, 4.271)	0.013		2.076 (1.102, 3.912)	0.024
Other		0.781 (0.270, 2.258)	0.648		0.725 (0.252.2.084)	0.551
Marital status	0.204		NI	0.138		NI
Married						
Unmarried						
Unknown						NI
Grade	0.108		NI	0.078		
Grade I/II						
Grade III/IV						
Unknown						
AJCC stage	<0.001		<0.001	<0.001		<0.001
IIIA		Reference			Reference	
IIIB		1.465 (0.645, 3.328)	0.361		1.541 (0.702, 3.380)	0.281
IV		4.837(2.548, 9.180)	<0.001		4.475(2.387, 8.389)	<0.001
Breast Subtype	<0.001		<0.001	<0.001		<0.001
Her2-/HoR+		Reference			Reference	
Her2+/HoR-		0.870 (0.330, 2.294)	0.778		0.820 (0.314, 2.143)	0.686
Her2+/HoR+		0.244 (0.070, 0.857)	0.028		0.225 (0.065, 0.785)	0.019
Triple Negative		3.387 (1.697, 6.762)	0.001		3.188 (1.630, 6.235)	0.001
Unknown		1.988 (0.655, 6.036)	0.225		1.897 (0.633, 5.685)	0.253
Surgery	0.843		0.307	0.738		0.254
Simple Mastectomy		Reference			Reference	
Radical mastectomy		1.493 (0.692, 3.222)			1.556 (0.728, 3.323)	
Chemotherapy	0.459		0.637	0.438		0.549
No/unknown		Reference			Reference	
Yes		1.641 (0.209, 12.882)			1.875 (0.241, 14.607)	
PMRT	0.013		0.030	0.006		0.013
No		Reference			Reference	
Yes		0.519 (0.287, 0.939)			0.480 (0.269, 0.859)	

**Notes.**

Abbreviation HoR Hormone receptor CSS cancerspecific survival OS overall survival NI not included in the multivariate survival analysis PMRT post mastectomy radiotherapy

## Discussion

To our knowledge, it is the first large population-based analysis to evaluate the survival benefit of PMRT in treating IBC patients, along with the application of PSM.A total of 293 eligible cases were analyzed in our study. We found that patients in the PMRT group had significant survival benefit after PSM. Additionally, PMRT was an independent favorable prognostic factor for CSS and OS. Further subgroup analysis revealed that nearly all subgroup population could benefit from PMRT.

More effective neoadjuvant therapy has enhanced the clinical response rate, and additionally, a considerable percentage of the previously unresectable subjects are proper for mastectomy (Brzezinska & Dixon, 2018; Chen et al., 2017; Yamauchi et al., 2009). Despite the incompletely validated survival benefit of PMRT, the advances in loco-regional control render PMRT on the thoracic wall and regional lymph nodes as an optional therapeutic option (Al-Hilli & Boughey, 2016; Chen et al., 2015). However, PMRT is underused in clinical practice. By utilizing the 1998 to 2011 National Cancer Data Base, a study conducted by MD Anderson Cancer Center revealed that 8273 females undergoing mastectomy for non-metastatic IBC (Loveland-Jones et al., 2016). Among those patients, over 30% did not undergo PMRT, mainly due to patients’ choice (86.4%), as well as other reasons including treatment at lower-volume centers, annual income, restricted coverage from medical insurance and distance between patient’s residence and radiotherapy facility (Arthur et al., 1999). Moreover, the non-receipt of PMRT rate for non-inflammatory but loco-regionally advanced breast cancer recorded in the literature was even higher, varying from approximately 35% to 50% (Chu et al., 2015; Shirvani et al., 2011).Therefore, this study is designed, aiming at that more patients and doctors can realize the significance of PMRT in IBC by confirming the survival benefits of PRMT.

At present, there are no established risk factors for IBC. However, multiple epidemiological studies have clarified the characteristics of IBC (Van Uden et al., 2015). The most important suspected risk factors associated with IBC include black race, body mass index(BMI), and age (Chang, Buzdar & Hursting, 1998). Additionally, Wu et al. found that breast cancer subtype was clinically useful to predict survival outcome in IBC. Patients with HoR-/HER2- subtype had significantly poorer OS compared to the other three subtypes. While patients with HoR+/HER2+ subtype harbored more favorableOS than the HoR+/HER2- subtype (Wu et al., 2019), which are consistent with our findings. However, age was not found to be a risk factor for prognosis in our study.

Our study found that radiotherapy is an independent prognostic risk factor for IBC. Some scholars have come to the same conclusion as ours. Diao et al. established and validated the prognostic model of IBC patients. From January 2004 to December 2013, 1,651 patients were enrolled in this study. Surgery, radiotherapy, and chemotherapy were all independent prognostic risk factors for IBC patients, indicating that multimodal comprehensive treatment could benefit IBC patients significantly (Diao et al., 2019). Similar results were also obtained in Pan X’s study (Pan et al., 2019). Comparing the two studies with ours, our enrollment was significantly smaller, mainly because our research time span was from 2010 to 2013.The main reason for choosing this time range is that the HER-2 factor was not available prior to 2010 in the SEER database. Breast cancer subtypes have been shown to be an independent prognostic risk factor for inflammatory breast cancer (Gonzalez-Angulo et al., 2007; Wu et al., 2019), so it is necessary to include the factor. However, such a short time range of enrollment also directly leads to less enrollment and shorter follow-up time in our study.

There are certain limitations to our research. First, as an observational study, the possibility of bias might be inevitable. Though the PSM method was used to decrease the bias resulting from unevenly-distributed measured covariates, it is unavoidable that unmeasured factors might cause bias. Second, there is a sizeable amount of missing data in the SEER dataset which can result in bias. Third, radiotherapy field design and dose may influence disease recurrence and survival outcome (Robertson et al., 2010); however, SEER does not record radiotherapy treatment details. In addition, the median follow-up time of 27 months in our study is too short to accurately reflect the long-term survival outcomes of IBC. Moreover, there are inaccuracies in the SEER database, with high rates of under-reporting for receipt of radiotherapy and chemotherapy (Jagsi et al., 2012; Noone et al., 2016). For this reason, we have adopted many methods to remedy these defects. Schoenfeld residual test was used to prove the proportional-hazard assumption of Cox model. Results revealed that all variables and the whole model were consistent with the hypothesis (all *P* > 0.05). In addition, we adopted the R software (version 3.6) making use of the “MICE” package for data imputation. Data with missing value (less than 20%) were imputed using the Multivariable Imputation by Chained Equations algorithm as described in Van Buuren & Groothuis-Oudshoorn (2011). Although obtaining more details is preferable, our aim is to demonstrate the general survival advantage of PMRT for IBC patients. Thus, the currently accessible data in the SEER dataset meet our research design and objectives.

## Conclusion

Our findings have validated the survival benefits of PMRT for IBC patients, which might optimize the present individualized therapeutic strategies for specific populations. Further prospective clinical trials are still needed to validate our findings.

##  Supplemental Information

10.7717/peerj.8512/supp-1 File S1 The plot of propensity score distribution score

10.7717/peerj.8512/supp-2 File S2 Raw data of included patients

## Additional Information and Declarations

### Data Availability

The following information was supplied regarding data availability: The raw measurements are available in the Supplemental Files.

## References

[ref-1] Al-Hilli Z, Boughey JC (2016). The timing of breast and axillary surgery after neoadjuvant chemotherapy for breast cancer. Chinese Clinical Oncology.

[ref-2] Arthur DW, Schmidt-Ullrich RK, Friedman RB, Wazer DE, Kachnic LA, Amir C, Bear HD, Hackney MH, Smith TJ, Lawrence Jr W (1999). Accelerated superfractionated radiotherapy for inflammatory breast carcinoma: complete response predicts outcome and allows for breast conservation. International Journal of Radiation Oncology, Biology, Physics.

[ref-3] Baldini E, Gardin G, Evagelista G, Prochilo T, Collecchi P, Lionetto R (2004). Long-term results of combined-modality therapy for inflammatory breast carcinoma. Clinical Breast Cancer.

[ref-4] Bartelink H, Rubens RD, Van der Schueren E, Sylvester R (1997). Hormonal therapy prolongs survival in irradiated locally advanced breast cancer: a European organization for research and treatment of cancer randomized phase III trial. Journal of Clinical Oncology.

[ref-5] Bourgier C, Pessoa EL, Dunant A, Heymann S, Spielmann M, Uzan C, Mathieu MC, Arriagada R, Marsiglia H (2012). Exclusive alternating chemotherapy and radiotherapy in nonmetastatic inflammatory breast cancer: 20 years of follow-up. International Journal of Radiation Oncology, Biology, Physics.

[ref-6] Brzezinska M, Dixon JM (2018). Inflammatory breast cancer: no longer an absolute contraindication for breast conservation surgery following good response to neoadjuvant therapy. Gland Surgery.

[ref-7] Budach W, Matuschek C, Bolke E, Dunst J, Feyer P, Fietkau R, Haase W, Harms W, Piroth MD, Sautter-Bihl ML, Sedlmayer F, Souchon R, Wenz F, Sauer R (2015). DEGRO practical guidelines for radiotherapy of breast cancer V: therapy for locally advanced and inflammatory breast cancer, as well as local therapy in cases with synchronous distant metastases. Strahlentherapie und Onkologie.

[ref-8] Chang S, Buzdar AU, Hursting SD (1998). Inflammatory breast cancer and body mass index. Journal of Clinical Oncology.

[ref-9] Chen LJ, Chung KP, Chang YJ, Chang YJ (2015). Ratio and log odds of positive lymph nodes in breast cancer patients with mastectomy. Surgical Oncology.

[ref-10] Chen H, Wu K, Wang M, Wang F, Zhang M, Zhang P (2017). A standard mastectomy should not be the only recommended breast surgical treatment for non-metastatic inflammatory breast cancer: a large population-based study in the Surveillance, Epidemiology, and End Results database 18. Breast.

[ref-11] Chu QD, Caldito G, Miller JK, Townsend B (2015). Postmastectomy radiation for N2/N3 breast cancer: factors associated with low compliance rate. Journal of the American College of Surgeons.

[ref-12] Cronin KA, Ries LA, Edwards BK (2014). The Surveillance, Epidemiology, and End Results (SEER) program of the national cancer institute. Cancer.

[ref-13] Dawood S, Merajver SD, Viens P, Vermeulen PB, Swain SM, Buchholz TA, Dirix LY, Levine PH, Lucci A, Krishnamurthy S, Robertson FM, Woodward WA, Yang WT, Ueno NT, Cristofanilli M (2011). International expert panel on inflammatory breast cancer: consensus statement for standardized diagnosis and treatment. Annals of Oncology.

[ref-14] Diao JD, Ma LX, Sun MY, Wu CJ, Wang LJ, Liu YL, Yang YJ (2019). Construction and validation of a nomogram to predict overall survival in patients with inflammatory breast cancer. Cancer Medicine.

[ref-15] Duggan MA, Anderson WF, Altekruse S, Penberthy L, Sherman ME (2016). The Surveillance, Epidemiology, and End Results (SEER) program and pathology: toward strengthening the critical relationship. American Journal of Surgical Pathology.

[ref-16] Gianni L, Eiermann W, Semiglazov V, Manikhas A, Lluch A, Tjulandin S, Zambetti M, Vazquez F, Byakhow M, Lichinitser M, Climent MA, Ciruelos E, Ojeda B, Mansutti M, Bozhok A, Baronio R, Feyereislova A, Barton C, Valagussa P, Baselga J (2010). Neoadjuvant chemotherapy with trastuzumab followed by adjuvant trastuzumab versus neoadjuvant chemotherapy alone, in patients with HER2-positive locally advanced breast cancer (the NOAH trial): a randomised controlled superiority trial with a parallel HER2-negative cohort. The Lancet.

[ref-17] Gonzalez-Angulo AM, Hennessy BT, Broglio K, Meric-Bernstam F, Cristofanilli M, Giordano SH, Buchholz TA, Sahin A, Singletary SE, Buzdar AU, Hortobagyi GN (2007). Trends for inflammatory breast cancer: is survival improving?. Oncologist.

[ref-18] Hance KW, Anderson WF, Devesa SS, Young HA, Levine PH (2005). Trends in inflammatory breast carcinoma incidence and survival: the surveillance, epidemiology, and end results program at the National Cancer Institute. Journal of the National Cancer Institute.

[ref-19] Howlader N, Chen VW, Ries LA, Loch MM, Lee R, DeSantis C, Lin CC, Ruhl J, Cronin KA (2014). Overview of breast cancer collaborative stage data items–their definitions, quality, usage, and clinical implications: a review of SEER data for 2004–2010. Cancer.

[ref-20] Jagsi R, Abrahamse P, Hawley ST, Graff JJ, Hamilton AS, Katz SJ (2012). Underascertainment of radiotherapy receipt in surveillance, epidemiology, and end results registry data. Cancer.

[ref-21] Kleer CG, Van Golen KL, Merajver SD (2000). Molecular biology of breast cancer metastasis. Inflammatory breast cancer: clinical syndrome and molecular determinants. Breast Cancer Research.

[ref-22] Little RJ, Rubin DB (2000). Causal effects in clinical and epidemiological studies via potential outcomes: concepts and analytical approaches. Annual Review of Public Health.

[ref-23] Loveland-Jones C, Lin H, Shen Y, Bedrosian I, Shaitelman S, Kuerer H, Woodward W, Ueno N, Valero V, Babiera G (2016). Disparities in the use of postmastectomy radiation therapy for inflammatory breast cancer. International Journal of Radiation Oncology, Biology, Physics.

[ref-24] Noone AM, Lund JL, Mariotto A, Cronin K, McNeel T, Deapen D, Warren JL (2016). Comparison of SEER treatment data with medicare claims. Medical Care.

[ref-25] Orecchia R (2018). Radiation therapy for inflammatory breast cancer. European Journal of Surgical Oncology.

[ref-26] Pan X, Yang W, Chen Y, Tong L, Li C, Li H (2019). Nomogram for predicting the overall survival of patients with inflammatory breast cancer: a SEER-based study. Breast.

[ref-27] Pattanayak CW, Rubin DB, Zell ER (2011). Propensity score methods for creating covariate balance in observational studies. Revista Espanola de Cardiologia.

[ref-28] Robertson FM, Bondy M, Yang W, Yamauchi H, Wiggins S, Kamrudin S, Krishnamurthy S, Le-Petross H, Bidaut L, Player AN, Barsky SH, Woodward WA, Buchholz T, Lucci A, Ueno NT, Cristofanilli M (2010). Inflammatory breast cancer: the disease, the biology, the treatment. CA: A Cancer Journal for Clinicians.

[ref-29] Shirvani SM, Pan IW, Buchholz TA, Shih YC, Hoffman KE, Giordano SH, Smith BD (2011). Impact of evidence-based clinical guidelines on the adoption of postmastectomy radiation in older women. Cancer.

[ref-30] Van Buuren S, Groothuis-Oudshoorn K (2011). MICE: multivariate imputation by chained equations in R. Journal of Statistical Software.

[ref-31] Van Uden DJ, Bretveld R, Siesling S, De Wilt JH, Blanken-Peeters CF (2017). Inflammatory breast cancer in the Netherlands; improved survival over the last decades. Breast Cancer Research and Treatment.

[ref-32] Van Uden DJ, Van Laarhoven HW, Westenberg AH, De Wilt JH, Blanken-Peeters CF (2015). Inflammatory breast cancer: an overview. Critical Reviews in Oncology/Hematology.

[ref-33] Wu SG, Zhang WW, Wang J, Dong Y, Sun JY, Chen YX, He ZY (2019). Inflammatory breast cancer outcomes by breast cancer subtype: a population-based study. Future Oncol.

[ref-34] Yamauchi H, Cristofanilli M, Nakamura S, Hortobagyi GN, Ueno NT (2009). Molecular targets for treatment of inflammatory breast cancer. Nature Reviews Clinical Oncology.

