# Supplementary figures and images for: Radiotherapy after mastectomy has significant survival benefits for inflammatory breast cancer: a SEER population-based retrospective study
Source: PeerJ. 2020 Feb 3;8:e8512. doi: 10.7717/peerj.8512 (PMC7003697; doi:10.7717/peerj.8512)

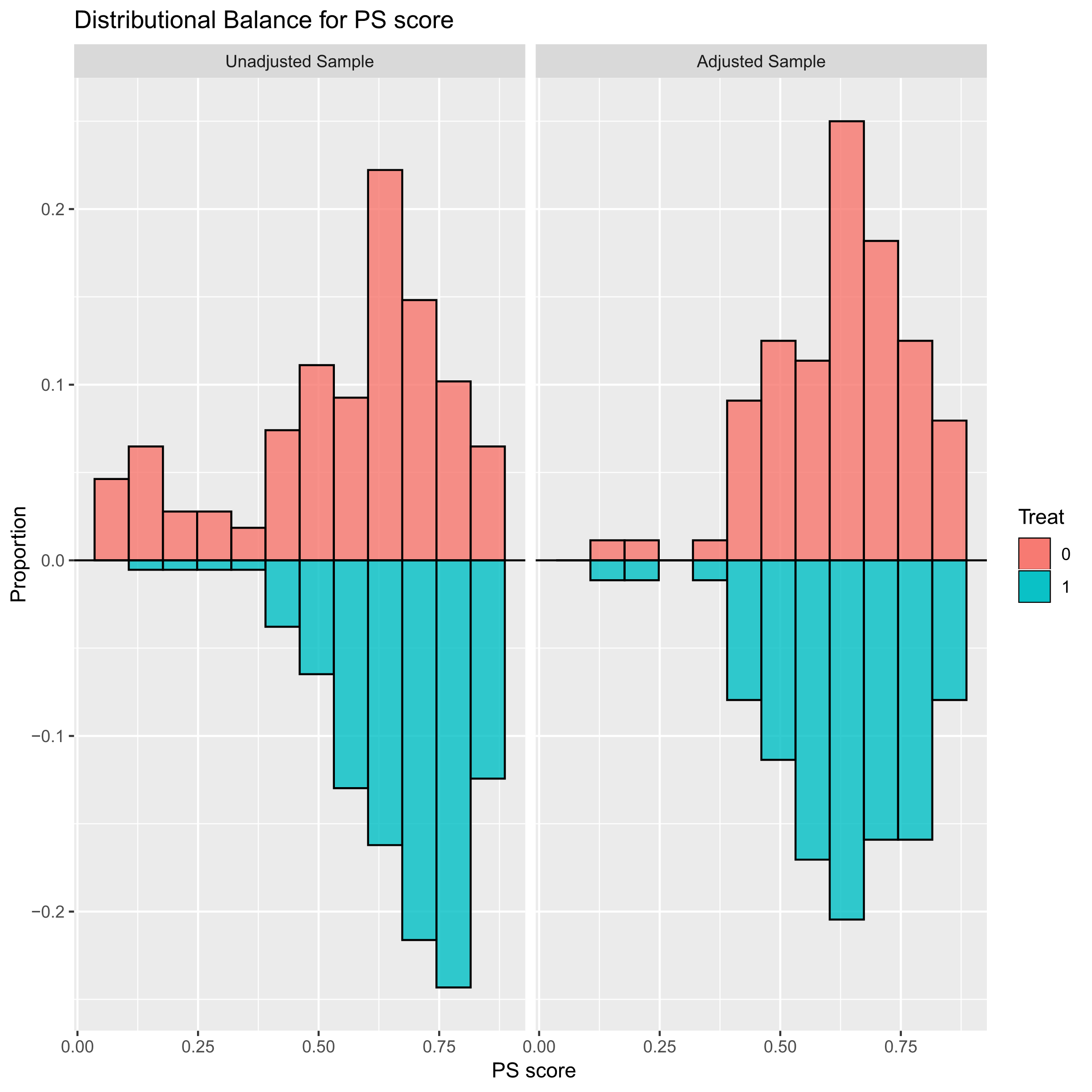

Supplement: File S1 [file peerj-08-8512-s001.png]
